# Draft genome sequence of the *Serratia liquefaciens* strain UNJFSC002, isolated from the soil of a potato field of the Bicentenaria variety

**DOI:** 10.1128/mra.00495-24

**Published:** 2025-02-25

**Authors:** Pedro M. Rodriguez-Grados, Carlos I. Arbizu, Gianmarco Castillo, Vladimir Camel, Sergio Conteras-Liza

**Affiliations:** 1 Departamento de Agronomía, Universidad Nacional José Faustino Sanchez Carrión, Lima, Peru; 2 Centro de Investigación en Germoplasma Vegetal y Mejoramiento Genético de Plantas (CIGEMP), Universidad Nacional Toribio Rodríguez de Mendoza de Amazonas (UNTRM), Amazonas, Peru; 3 Instituto de Investigación para el Desarrollo Sustentable de Ceja de Selva (INDES-CES), Universidad Nacional Toribio Rodríguez de Mendoza de Amazonas, Amazonas, Peru; 4 Facultad de Ingeniería y Ciencias Agrarias, Universidad Nacional Toribio Rodríguez de Mendoza de Amazonas (UNTRM), Amazonas, Peru; 5 Escuela Profesional de Ingeniería Ambiental, Universidad Cesar Vallejo, Lima, Peru; University of Strathclyde, Glasgow, United Kingdom

**Keywords:** *Serratia*, plant-microbe interactions, gene sequencing, functional annotation

## Abstract

In this study, we present the draft genome assembly of the *Serratia liquefaciens* strain UNJFSC002, isolated from the experimental potato field of the Bicentenaria variety at the Universidad Nacional José Faustino Sánchez Carrión, Lima, Peru.

## ANNOUNCEMENT

The genus *Serratia* inhabits a variety of environments, including soil, bodies of water, and food products (1). Bacteria of the genus *Serratia* are gram negative and are widely distributed worldwide. They can thrive in various environments, such as water, soil, and the digestive tracts of multiple animals (2).

One hundred grams of soil was collected 4 cm from the root of the most vigorous Bicentenaria variety potato plant in the experimental field of the Universidad Nacional José Faustino Sánchez Carrión (UNJFSC) in Lima, Peru (11°07′24.7″S;77°36′30.5″ W), the soil was sieved, and 10 g was taken to dilute in 90 mL of sterile saline solution (NaCl); serial dilutions were performed in nutrient broth (Himedia, India; catalog number: M002) and incubated at 30°C for 48 hours. Then, the 10^6^ dilution was plated on a petri dish with solid TSA medium (Himedia, India; catalog number: M1968). The grown colony underwent five subcultures to obtain a pure strain, in addition to observing its structure on a petri dish and with Gram staining. To extract the genomic DNA, the strain was transferred to the nutrient broth and incubated at 30°C for 18 hours at 150 rpm (to have approximately 2 × 10⁹ cells). Then, the EZ-10 Spin Column Bacterial Genomic DNA Miniprep Kit (Bio Basic, Canada) was used following the manufacturer’s protocol. Subsequently, the library preparation and sequencing were performed by Macrogen Inc. (Seoul, Republic of Korea) using Illumina NovaSeq6000 150 paired-end (150 × 2 bp) sequencing with the Illumina Nextera DNA XT library.

The genome sequencing generated 11,235,041 read pairs, representing a genome coverage of approximately 150×. The quality of the reads was assessed using FastQC version 0.12.1 (3). The reads were trimmed and filtered (Phred *Q* > 25) using Trimmomatic version 0.36 (4) using default parameters to remove Illumina adapters and eliminate low-quality bases. *De novo* assembly was performed using Unicycler version 0.5.0 (5), testing various k-mers ranging from 27 to 127. Assembly statistics were obtained using QUAST version 5.2.0 (6). Additionally, to estimate the completeness and consistency of the assembled genome, Benchmarking Universal Single-Copy Orthologs (BUSCO) version 5.7.0 (7) was used with the data set gammaproteobacteria_odb10 (https://busco.ezlab.org/frames/bact.htm), as well as the Microbial Genome Atlas (MiGA) webserver version 2.0 (8), showing 100% completeness of the strain *Serratia liquefaciens* ATCC 27592 GCF 000422085 1. The genome JBAKHJ000000000 available on NCBI was annotated by PGAP version 6.6 (9), but a local Prokka version 1.14.5 (10) annotation estimated 4,792 protein-coding genes in the assembly (Table 1). Additionally, 80 tRNA, 1 tmRNA, and 44 contigs were detected in the genome. Additionally, the MiGA tool classified the strain based on average nucleotide identity (ANI), as most similar to *Serratia liquefaciens* ATCC 27592 with accession number CP014017 and with 98.97% ANI.

**TABLE 1:** Quality statistics of *S. liquefaciens* strain UNJFSC 002

Feature	Result
Sequence reads	
Read pairs	11,235,041
Assembly Prokka	
DNA genome size (bp)	5,199,434
Contigs	44
Coverage	33
G + C content (%)	55.33
Protein-coding genes	5,019
tmRNA genes	1
tRNA genes	75
Contigs *N*_50_/*L*_50_ (bp)	376/7
MiGA (%)	
Completeness	100.00
Contamination	1.9
Quality	90.5
No. of BUSCOs	
Complete	439
Duplicate	1
Fragmented	1
Missing	0

## Data Availability

The sequence reads generated in this study have been submitted to the Sequence Read Archive (SRA) under the accession number SRR28705404. The assembled genome has been deposited to NCBI GenBank under the accession number JBAKHJ000000000, the BioProject accession number PRJNA1076138, and the BioSample accession number SAMN39932967. The PROKKA annotation files have been published on Zenodo (DOI: https://doi.org/10.5281/zenodo.11068178).
